# Pre-Stroke Loop Diuretics and Anemia in Elderly Patients Are Associated Factors of Severe Renal Dysfunction at the Time of Acute Stroke Onset

**DOI:** 10.3390/jcdd10090405

**Published:** 2023-09-19

**Authors:** Takahisa Mori, Tetsundo Yano, Kazuhiro Yoshioka, Yuichi Miyazaki

**Affiliations:** Department of Stroke Treatment, Shonan Kamakura General Hospital, Kamakura City 247-8533, Japan; liptetu2008@yahoo.co.jp (T.Y.); y.kazuhiro12@icloud.com (K.Y.); y.miyazaki@me.com (Y.M.)

**Keywords:** anemia, aspirin, calcium channel blockers, loop diuretics, renal dysfunction, sex, stroke

## Abstract

Background: Severe renal dysfunction (SRD), an advanced stage of chronic kidney disease (CKD), can limit the treatment options for acute stroke (AS) patients. Therefore, it is important to investigate the associated factors of SRD in AS patients to inhibit CKD progression to SRD before AS. Sex differences exist in the renal function. Therefore, we investigated the frequency of SRD and its associated factors among AS patients by sex. Methods: Our cross-sectional study included patients admitted within 24 h of AS onset between 2013 and 2019 with available pre-stroke medication information. We used the Cockcroft–Gault equation for calculating the creatinine clearance (Ccr) and defined SRD as a Ccr < 30 mL/min. We performed multivariable logistic regression analysis to identify the independent factors associated with SRD. Results: Out of 4294 patients, 3472 matched our criteria. Of these, 1905 (54.9%) were male, with median ages of 75 and 81 years for males and females, respectively. The frequency of SRD was 9.7% in males and 18.7% in females. Loop diuretics and anemia were associated factors of SRD. Conclusions: Pre-stroke loop diuretics and anemia in elderly patients were associated factors of SRD in both sexes. Individualized drug therapy and anemia management are essential to prevent SRD.

## 1. Introduction

The renal function is often impaired in acute stroke (AS) patients due to chronic kidney disease (CKD), a common condition associated with vascular risk factors, such as hypertension, diabetes, dyslipidemia, and smoking [1,2,3]. CKD, which affects about 20%–46% of AS patients [4], increases the risk of cardiovascular events, such as heart disease and stroke [5,6,7,8,9,10,11,12]. CKD can progress to severe renal dysfunction (SRD), defined as a creatinine clearance (Ccr) of less than 30 mL/min [13,14,15,16,17,18,19,20]. SRD complicates the treatment during the AS stage and has significant implications for AS management and prognosis. SRD limits the pharmacological options due to the increased risk of adverse effects [13,14,15,16,17,18,19,20] during the acute stroke (AS) stage and increases the risk of in-hospital mortality after the treatment of the tissue plasminogen activator [21]. Moreover, SRD may limit the eligibility and feasibility of mechanical thrombectomy in patients with SRD [22,23].

Therefore, it is important to investigate the incidence and associated factors of SRD in AS patients to inhibit CKD progression to SRD before AS, optimize the management of SRD during the AS stage, and improve patient outcomes. Sex differences should be considered in the renal function and CKD progression, as women tend to have lower serum creatinine levels and higher Ccr levels than men due to gonadal hormones and lower muscle mass and creatinine production [24,25]. In this cross-sectional study, we aimed to examine the frequency of SRD in AS patients and its associated factors by sex.

## 2. Materials and Methods

### 2.1. Patients

We conducted a cross-sectional study based on an institutional stroke registry database with the consecutive enrollment of patients. We included patients who met the following criteria: (1) admission within 24 h of AS onset between January 2013 and March 2019 and (2) available pre-stroke medication information. We excluded patients whose serum creatinine levels or body weights were not measured upon arrival.

### 2.2. Materials

We collected data upon arrival on age; sex; body weight; pre-stroke medications; serum levels of albumin, creatinine, glucose, glycated hemoglobin A1c, lipids, and c-reactive protein; the pre-stroke modified Rankin scale; and stroke subtypes (ischemia or hemorrhage). Lipids were total cholesterol, low-density lipoprotein cholesterol, high-density lipoprotein cholesterol, and triglycerides. Antihypertensives were alpha blockers, alpha–beta blockers, beta blockers, angiotensin-converting enzyme inhibitors (ACEis), angiotensin receptor blockers (ARBs), L-type calcium channel blockers (CCBs), T-type or N-type CCBs, and mineralocorticoid receptor antagonists (MRAs). Diabetes medications were alpha-glucosidase inhibitors (alpha-GIs), dipeptidyl peptidase 4 inhibitors, biguanides, sodium–glucose cotransporter 2 inhibitors, insulin secretagogues (ISs), and long-acting insulin analogs. Diuretics were loop or thiazide. Antiplatelets were aspirin or non-aspirins. Anticoagulants were warfarin or direct-acting oral anticoagulants (DOACs). We did not include past medical history for analysis as we could not collect it accurately.

### 2.3. Procedures

We used the Cockcroft–Gault equation for calculating the Ccr using age, body weight, the serum creatinine level, and sex [26]. Ccr is correlated with the glomerular filtration rate [27], and a Ccr < 30 mL/min limits pharmacological treatments [13,14,15,16,17,18,19,20]. Therefore, we defined SRD as a Ccr < 30 mL/min and investigated the frequency of SRD in AS patients and the significant factors associated with SRD, especially pre-stroke medications, by sex.

### 2.4. Statistical Analysis

We tested the normality of the continuous variables using the Shapiro–Wilk test. We expressed normally distributed continuous variables as mean ± standard deviation and non-normally distributed continuous variables as median and interquartile range. The Wilcoxon rank-sum test was used to compare unpaired groups of SRD (Ccr < 30 mL/min) and non-SRD (Ccr ≥ 30 mL/min). The chi-square test was used to compare the categorical variables between the two groups. When the cell count was below 5, Fisher’s exact test was performed.

For multivariable analysis, we used variables with significant differences between SRD and non-SRD by sex and used a dummy variable (1 or 0) to represent categorical data, such as data on medications (1 for use, 0 for no use). We performed the multivariable logistic regression analysis to identify independent factors associated with SRD upon admission by sex. We excluded age, body weight, and the serum creatinine level from the multivariable analysis, as they are components of the Cockcroft–Gault equation and strongly correlated with Ccr. If some pre-stroke medications were independently related to SRD, then we performed a subgroup analysis to examine the association among the medications and between the combined use of the medications and SRD.

A *p*-value < 0.05 was considered statistically significant. We used JMP software (version 17.1; SAS Institute, Cary, NC, USA) for all statistical analyses. One author (T.M.) had full access to all the data in the study and took responsibility for their integrity and the data analysis.

## 3. Results

Of 4294 patients with AS, 3472 matched our inclusion and exclusion criteria. The baseline characteristics of the patients stratified by sex are summarized in Table 1, which shows that 1905 (54.9%) were male, with median ages of 75 years for males and 81 years for females, and the frequency of SRD was 9.7% in males and 18.7% in females. The SRD occurred in females more frequently at AS onset. The medications of males and females before AS are summarized in supplementary Table S1. The association of pre-stroke variables with renal dysfunction is summarized by sex in supplementary Tables S2 and S3.

Multivariable logistic regression analysis using variables with significant differences for males identified loop diuretics, aspirin, L-type CCBs, alpha–beta blockers, alpha blockers, anemia, and a low albumin level as independent factors that increased the risk of SRD (Table 2). Moreover, the receiver-operating-characteristics curve of the logistic regression analysis revealed that the cut-off values of hemoglobin and albumin for predicting a Ccr < 30 mL/min in male patients were ≤130 g/L (13.0 g/dL) and ≤38 g/L (3.8 mg/dL), respectively (Supplementary Tables S4 and S5, Supplementary Figures S1 and S2). Multivariable logistic regression analysis using variables with significant differences for females identified loop diuretics, anemia, and MRAs as independent factors that increased the risk of SRD, while DOACs were factors that decreased the risk of SRD (Table 3). Moreover, the receiver-operating-characteristics curve of the logistic regression analysis revealed that the cut-off value of hemoglobin for predicting a Ccr < 30 mL/min in female patients was ≤124 g/L (12.4 g/dL) (Supplementary Table S6, Supplementary Figure S3).

Subgroup analysis in male patients revealed that, among 150 using loop diuretics, the proportion of patients using aspirin, MRAs, alpha–beta blockers, beta blockers, ARBs, ACEis, DOACs, or warfarin was significantly higher than among those not using loop diuretics (Supplemental Table S7). Loop diuretics were often used with MRAs (Supplemental Table S7). However, the frequency of SRD in MRA users was not significantly high, although that in loop diuretic users was significant (Supplemental Table S2). The prevalence of SRD was 52% (13 out of 25) among male patients using loop diuretics, aspirin, and ARBs (Supplemental Table S8). Loop diuretics in male patients were associated with other medications used for heart failures, such as MRAs, alpha–beta blockers, beta blockers, ARBs, ACEis, DOACs, or warfarin. However, only loop diuretics and alpha–beta blockers were independently associated with SRD.

Subgroup analysis in female patients revealed that, among 168 using loop diuretics, the proportion of patients using aspirin, MRAs, alpha–beta blockers, beta blockers, ARBs, DOACs, or warfarin was significantly higher than among those not using loop diuretics (Supplemental Table S9).

Loop diuretics were often used with MRAs (Supplementary Table S9). The frequency of SRD in loop diuretic or MRA users was significantly high (Supplementary Table S3). The prevalence of SRD was 52% (13 out of 25) among male patients using loop diuretics, aspirin, and ARBs (Supplemental Table S8). The prevalence of SRD was 53.3% (8 out of 15) among female patients using loop diuretics, aspirin, and ARBs (Supplemental Table S10).

Loop diuretics in female patients were associated with other medications used for heart failures, such as MRAs, alpha–beta blockers, beta blockers, ARBs, DOACs, or warfarin. However, only loop diuretics and MRAs were independent associated factors of SRD.

## 4. Discussion

Our findings suggest that pre-stroke loop diuretics and anemia in elderly patients increase the risk of SRD at AS onset in both sexes. Moreover, sex differences exist in the factors associated with SRD, implying that elderly male and female patients with CKD may require different preventive and therapeutic strategies to delay SRD. Loop diuretics are commonly prescribed medications for congestive heart failure, suggesting the importance of considering cardio–renal anemia (CRA) syndrome [28] in clinical practice and the need for individualized drug therapy and anemia management by sex to prevent SRD.

CRA syndrome suggests an interaction between anemia, congestive heart failure, and chronic kidney insufficiency, contributing to the deterioration of the anemia cardiac and renal function [28]. Heart failure impairs the renal function, which causes anemia, which, in turn, exacerbates heart failure. This cycle continues unless interrupted. Moreover, heart failure is also a risk factor for all stroke subtypes [29]. Therefore, CRA syndrome should be detected and treated early. Even mild anemia, indicated by Hb < 13.8 g/dL, increases the risk for progression to end-stage renal disease [30]. Additionally, subjects with low hematocrits (<40% for men and <35% for women) have a significantly increased risk of end-stage renal disease [31]. Therefore, it is essential to treat anemia before CRA syndrome progresses.

The early initiation of erythropoietin in pre-dialysis patients with non-severe anemia significantly slows the progression of renal disease and delays the initiation of renal replacement therapy [32]. Moreover, treating anemia with erythropoietin-stimulating agents improves energy and the physical function in non-dialysis CKD patients [33]. However, the optimal timing of initiating anemia treatment remains controversial. Maintaining higher Hb levels (11.0 ≤ Hb < 13.0 g/dL) with darbepoetin alfa has been shown to better preserve the renal function in patients with CKD not on dialysis compared to maintaining lower Hb levels (9.0 ≤ Hb < 11.0 g/dL) [34]. Therefore, maintaining high Hb levels is recommended before the progression of anemia. However, a high target-hemoglobin level using erythropoietin-stimulating agents may not always benefit patients with CRA syndrome. A target hemoglobin level of 13.5 g/dL, compared with 11.3 g/dL, was associated with an increased risk of cardiovascular events or stoke and no incremental improvement in the quality of life [35,36]. Additionally, the early complete correction of anemia using erythropoietin-stimulating agents does not reduce the risk of cardiovascular events [37]. Therefore, in the real-world clinical practice of clinic physicians, initiating treatment of patients with Hb > 130 g/L and a renal function of Ccr ≥ 30 mL/min may be delayed and challenging, as our study found that Hb cut-off values of 130 g/L and 124 g/L were associated with predicting a Ccr < 30 mL/min in our male and female patients, respectively. This delay in treatment initiation can potentially lead to the progression of CRA syndrome by exacerbating the anemia cardiac and renal function. CKD patients should have their declining hemoglobin levels detected early. It is essential to prevent it safely by using alternative methods rather than erythropoietin-stimulating agents when hemoglobin levels drop. Renal failure or heart failure patients need to ensure adequate sodium restriction [38,39] if their salt intake is not reduced enough to interrupt the cycle of CRA syndrome.

Loop diuretics, MRAs, alpha–beta blockers, beta blockers, ARBs, or ACEis are common medications for heart failure. Among 150 male patients using loop diuretics in our study, the proportion of these patients using any of the other five medications was significantly high (Supplemental Table S7). This suggests that congestive heart failure was likely associated with the prescription of these six medications. Therefore, heart failure could be a confounder of renal failure, as it is a risk factor. The univariable analysis revealed that loop diuretics were significant factors of SRD, but MRAs or ACEis were not (Supplemental Table S2). Moreover, loop diuretics have been reported to exacerbate the renal function [40]. Therefore, loop diuretics and alpha–beta blockers were associated factors of SRD, although the multivariable logistic analysis did not adjust for the history of heart failure.

However, the multivariable logistic analysis in male patients revealed that alpha blockers were significant factors for SRD. The doxazosin arm, an alpha blocker, was terminated early in the ALLHAT trial when the trial’s safety and monitoring board noted a two-fold higher incidence of congestive heart failure in patients receiving doxazosin than in those receiving chlorthalidone [41,42]. Therefore, heart failure may have been an intermediate factor between alpha blockers and SRD.

Furthermore, L-type CCBs are common medications for hypertension. However, the multivariable logistic analysis in male patients revealed that L-type CCBs were significant factors of SRD. In terms of renal-protective effects, L-type CCBs, such as amlodipine, are inferior to N-type or T-type CCBs [41,42,43,44]. Therefore, L-type CCBs could be risk factors for SRD.

Nonsteroidal anti-inflammatory drugs (NSAIDs), diuretics, and renin–angiotensin system inhibitors (RASis) are common medications for ischemic cardiovascular diseases and heart failure. However, their combined use, the triple whammy, increases the risk of acute kidney injury [45,46]. NSAIDs are widely used by older people, who also take diuretics for heart failure. The adverse renal effects of NSAIDs are reported, as they inhibit the prostaglandin-mediated dilation of the afferent renal arteriole. The risk of acute kidney injury is further increased by the combined use of NSAIDs with diuretics and RASis. In our male patients, loop diuretics and aspirin (an NSAID) were independent factors that increased the risk of SRD. The prevalence of SRD was high (52%) among male patients using the triple whammy, such as loop diuretics, aspirin, and ARBs (Supplemental Table S8).

Among 168 female patients using loop diuretics, the proportion of these patients using any MRAs, alpha–beta blockers, beta blockers, or ARBs was significantly high (Supplemental Table S9). This suggests that congestive heart failure was likely associated with the prescription of these five medications. Heart failure could be a confounder for renal failure as well. The univariable analysis revealed that loop diuretics were significant factors of SRD, but ACEis were not (Supplemental Table S3). Therefore, loop diuretics and MRAs were associated factors of SRD in female patients, although the multivariable logistic analysis did not adjust for the history of heart failure. Aspirin was not an independent associated factor of SRD in female patients. However, the prevalence of SRD was high (53.3%) among female patients using the triple whammy (Supplemental Table S10).

DOACs have been reported to have anti-inflammatory activity and vascular protection [47,48], suggesting consistency with our finding that DOACs were inversely associated with SRD in females.

If adverse effects due to medications exacerbate the renal function, then CRA syndrome progresses. Our study highlights the importance of considering CRA syndrome in patients with multiple vascular risk factors and suggests the need for individualized drug and anemia management to prevent SRD and improve the outcomes of AS patients.

All our patients had received medications from the clinic physicians, not from the nephrologists before AS onset. Clinic physicians need to be aware of the magnitude of the renal risk associated with loop diuretics, NSAIDs, or the triple whammy and pay attention to the renal function of their patients. Therefore, they should regularly check the renal function via blood tests in elderly patients and consider switching or modifying medications. Loop diuretics, aspirin, alpha blockers, alpha–beta blockers, and/or L-type CCBs are candidates for switching for elderly male patients, and loop diuretics and/or MRAs are candidates for elderly female patients. Sodium–glucose co-transporter 2 inhibitors (SGLT2is) have been reported to reduce the risk of CKD progression, cardiovascular death, or hospitalization for heart failure [49,50,51,52,53]. However, the proportion of SGLT2i users was very low compared to that of loop diuretics users in our patients (supplemental Table S1). Therefore, a possible treatment option for clinic physicians is to switch loop diuretics to SGLT2is for heart failure or CKD in both sexes. Moreover, it is an option to switch L-type CCBs to N-type or T-type CCBs in male patients. Additionally, anemia and hypoalbuminemia should be promptly detected and safely managed in both sexes to prevent them from dropping.

Our study has several limitations. Firstly, the cross-sectional design may introduce reverse causality into the relationships of SRD with pre-stroke medications, anemia, and DOACs. Heart failure is likely to be a confounder for SRD; however, the multivariable logistic analysis was not adjusted for the presence of heart failure. Second, the study population was mainly elderly Japanese, which limits the generalizability of the study outcomes to non-Japanese populations due to potential racial differences in medication efficacy. Third, the history of drugs may have been misclassified, introducing information bias. Fourth, there might be some unknown confounders for SRD. Fifth, we did not collect data on basic demographic information (education, income, marriage), lifestyles (smoking, drinking, physical activity, energy intake, salt intake, carbohydrate intake, fat intake, percentages of saturated fatty acids, n-6 polyunsaturated fatty acids, and n-3 polyunsaturated fatty acids), and family history of stroke, which could have biased the results. Therefore, a prospective study is warranted to confirm our findings.

## 5. Conclusions

Pre-stroke loop diuretics and anemia in elderly patients were associated factors of SRD at the time of AS onset in both sexes. Sex differences in the factors associated with SRD imply that elderly male and female patients with CKD may require different preventive and therapeutic strategies to delay SRD. Individualized drug therapy and anemia management are essential to inhibit SRD for elderly male and female patients. Our findings provide a basis for developing effective preventive strategies for SRD. Further prospective studies are warranted to validate our findings and to optimize individualized drug therapy and anemia management in elderly CKD patients for different sexes.

## Figures and Tables

**Table 1:** Characteristics of males and females upon admission.

Variables	Male Sex	Female Sex
	n = 1905	n = 1567
Ccr < 30 mL/min upon admission, n (%)	185 (9.7%)	293 (18.7%)
Age, years	75, 66.5–82	81, 72–87
Modified Rankin Scale before the acute stroke	0, 0–2	0, 0–3
Ischemia, n (%)	1517 (79.6%)	1218 (77.7%)
Body mass index, kg/m^2^	22.9, 20.8–25.0	20.9, 18.7–23.6
Height, cm	165, 161–170	151.4, 148–156
Body weight, kg	62.9, 55.9–70	48.4, 41.5–55
Albumin, g/L	40, 37–43	40, 36–43
Hemoglobin, g/L	141, 128–152	129, 117–1139
Glucose, mmol/L	6.88, 5.83–8.66	6.61, 5.77–8.27
Glycated hemoglobin A1c, %	5.8, 5.5–6.4	5.8, 5.5–6.2
Low-density lipoprotein cholesterol, mmol/L	2.81, 2.22–3.40	3.19, 2.58–3.86
High-density lipoprotein cholesterol, mmol/L	1.35, 1.11–1.66	1.56, 1.29–1.90
Triglycerides, mmol/L	1.12, 0.78–1.76	1.04, 0.77–1.48
Creatinine, µmol/L	82.2, 70.7–100.3	61.9, 53.0–73.4
C-reactive protein, µg/L	1200, 500–3900	1200, 500–4700

All values except for categorical data are represented as median and interquartile ranges. Ccr, creatinine clearance; n, number.

**Table 2:** Multivariable logistic regression analysis for Ccr < 30 mL/min in male patients.

Variables	OR 95% CI	*p*-Value	AUC
		<0.0001	0.884
Hemoglobin, g/L	0.95, 0.94–0.96	<0.0001	
Loop diuretics (1 for use, 0 for no use)	3.57, 2.21–5.76	<0.0001	
Modified Rankin scale before the acute stroke	1.21, 1.07–1.36	0.0021	
Alpha blockers (1 for use, 0 for no use)	3.93, 1.48–9.93	0.0046	
Albumin, g/L	0.95, 0.91–0.99	0.0123	
Aspirin (1 for use, 0 for no use)	1.66, 1.01–2.49	0.0150	
L-type CCBs (1 for use, 0 for no use)	1.61, 1.07–2.40	0.0209	
Alpha–beta blockers (1 for use, 0 for no use)	1.77, 1.04–2.97	0.0318	
Beta blockers (1 for use, 0 for no use)	1.70, 0.90–3.09	0.0902	
C-reactive protein, μg/L	1.00, 0.99–1.00	>0.100	
Biguanides (1 for use, 0 for no use)	1.77, 0.79–3.86	>0.100	
Alpha-glucosidase inhibitors (1 for use, 0 for no use)	1.60, 0.68–3.53	>0.100	
Low-density lipoprotein cholesterol, mmol/L	1.13, 0.90–1.40	>0.100	
Antiplatelets other than aspirin (1 for use, 0 for no use)	0.86, 0.51–1.42	>0.100	
DPP4is (1 for use, 0 for no use)	0.85, 0.45–1.53	>0.100	
Triglycerides, mmol/L	1.05, 0.84–1.28	>0.100	
ARB (1 for use, 0 for no use)	1.09, 0.72–1.63	>0.100	
Warfarin (1 for use, 0 for no use)	0.90, 0.47–1.65	>0.100	
High-density lipoprotein cholesterol, mmol/L	1.03, 0.64–1.64	>0.100	
Verapamil (1 for use, 0 for no use)	1.02, 0.27–3.40	>0.100	

ARBs, angiotensin receptor blockers; AUC, area under the curve; CCBs, calcium channel blockers; CI, confidence interval; DPP4is, dipeptidyl peptidase 4 inhibitors; OR, odds ratio; p, probability.

**Table 3:** Multivariable logistic regression analysis for Ccr < 30 mL/min in female patients.

Variables	OR 95% CI	*p*-Value	AUC
		<0.0001	0.809
Hemoglobin, g/L	0.96, 0.95–0.97	<0.0001	
Loop diuretics (1 for use, 0 for no use)	4.05, 2.67–6.17	<0.0001	
Modified Rankin scale before the acute stroke	1.26, 1.15–1.38	<0.0001	
DOACs (1 for use, 0 for no use)	0.28, 0.09–0.67	0.0031	
MRAs (1 for use, 0 for no use)	1.72, 1.01–2.90	0.0440	
ARBs (1 for use, 0 for no use)	1.40, 1.00–1.95	0.0496	
Alpha–beta blockers (1 for use, 0 for no use)	1.48, 0.87–2.55	>0.100	
Glycated hemoglobin A1c, %	0.91, 0.80–1.04	>0.100	
Low-density lipoprotein cholesterol, mmol/L	0.89, 0.75–1.04	>0.100	
High-density lipoprotein cholesterol, mmol/L	1.26, 0.89–1.78	>0.100	
Albumin, g/L	0.98, 0.94–1.01	>0.100	
Verapamil (1 for use, 0 for no use)	0.53, 0.18–1.39	>0.100	
Beta blockers (1 for use, 0 for no use)	1.34, 0.77–2.29	>0.100	
Aspirin (1 for use, 0 for no use)	1.18, 0.78–1.77	>0.100	
Warfarin (1 for use, 0 for no use)	1.10, 0.64–1.87	>0.100	
Alpha blockers (1 for use, 0 for no use)	1.31, 0.44–3.55	>0.100	
C-reactive protein, μg/L	0.99, 0.99–1.00	>0.100	

ARBs, angiotensin receptor blockers; AUC, area under the curve; CI, confidence interval; DOACs, direct-acting oral anticoagulants; MRAs, mineralocorticoid receptor antagonists; OR, odds ratio; p, probability.

## Data Availability

The datasets analyzed during the current study are available from the corresponding author upon reasonable request.

## Supplementary Materials

The following supporting information can be downloaded at https://www.mdpi.com/article/10.3390/jcdd10090405/s1, Table S1: Pre-stroke medications of males and females; Table S2: Association of pre-stroke variables with renal dysfunction in male patients; Table S3: Association of pre-stroke variables with severe renal dysfunction in female patients; Table S4: The cut-off value of hemoglobin for predicting Ccr < 30 mL/min in male patients using receiver-operating-characteristics curve of logistic regression analysis; Table S5: The cut-off value of albumin for predicting Ccr < 30 mL/min in male patients using receiver-operating-characteristics curve of logistic regression analysis; Table S6: The cut-off value of hemoglobin for predicting Ccr < 30 mL/min in female patients using receiver-operating-characteristics curve of logistic regression analysis; Table S7: Association of loop diuretic use with other heart-disease-relevant medications in male patients: the contingency table analysis; Table S8: Association of triple whammy with renal function in male patients; Table S9: Association of loop diuretic use with other heart-disease-relevant medications in female patients: the contingency table analysis; Table S10: Association of triple whammy with renal function in female patients; Figure S1: Receiver-operating-characteristics curve of hemoglobin for predicting Ccr < 30 mL/min in male patients. Area under the curve is 0.853; Figure S2: Receiver-operating-characteristics curve of albumin for predicting Ccr < 30 mL/min in male patients. Area under the curve is 0.735; Figure S3: Receiver-operating-characteristics curve of hemoglobin for predicting Ccr < 30 mL/min in female patients. Area under the curve is 0.727.
