# Supplementary material for: Pre-Stroke Loop Diuretics and Anemia in Elderly Patients Are Associated Factors of Severe Renal Dysfunction at the Time of Acute Stroke Onset
Source: J Cardiovasc Dev Dis. 2023 Sep 19;10(9):405. doi: 10.3390/jcdd10090405 (PMC10532343; doi:10.3390/jcdd10090405)
Supplement: Supplementary file 1 [file jcdd-10-00405-s001.zip › jcdd-2576359-supplementary.pdf]

Supplementary File

Table S1 Pre-stroke medications of males and females

Variables	Male Sex	Female Sex
	n=1905	n=1567
DPP4is, n (%)	235 (12.3%)	129 (8.2%)
Biguanide, n (%)	121 (6.4%)	57 (3.6%)
Alpha-GIs, n (%)	59 (3.1%)	26 (1.7%)
Insulin secretagogues, n (%)	125 (6.6%)	65 (4.1%)
Long-acting insulin analog, n (%)	51 (2.7%)	25 (1.6%)
SGLT2is, n (%)	14 (0.7%)	5 (0.3%)
Loop diuretics, n (%)	150 (7.9%)	168 (10.7%)
Thiazide diuretics, n (%)	40 (2.1%)	34 (2.2%)
MRAs, n (%)	85 (4.5%)	107 (6.8%)
ARBs, n (%)	447 (23.6%)	381 (24.4%)
L-type CCBs, n (%)	432 (22.7%)	350 (22.3%)
N-type or T-type CCBs, n (%)	80 (4.2%)	74 (4.7%)
Verapamil, n (%)	24 (1.3%)	34 (2.2%)
Diltiazem, n (%)	19 (1.0%)	16 (1.0%)
ACEis, n (%)	136 (7.1%)	84 (5.4%)
Alpha-blockers, n (%)	35 (1.8%)	25 (1.6%)
Alpha-beta blockers, n (%)	165 (8.7%)	111 (7.1%)
beta-blockers, n (%)	115 (6.0%)	111 (7.1%)
Statin, n (%)	417 (21.9%)	332 (21.2%)
Warfarin, n (%)	129 (6.8%)	108 (6.9%)
DOACs, n (%)	104 (5.5%)	73 (4.7%)
Aspirin, n (%)	353 (18.5%)	212 (13.5%)
Antiplatelets other than aspirin, n (%)	244 (12.8%)	131 (8.4%)

Alpha GIs, alpha glucosidase inhibitors; ARBs, angiotensin receptor blockers; ACEis, angiotensin-converting enzyme inhibitors; AS, acute stroke; CCBs, calcium channel blockers; DOACs, direct-acting oral anticoagulants; DPP4is, dipeptidyl peptidase 4 inhibitors; MRAs, mineralocorticoid receptor antagonists; P, probability; SGLT2i, sodium-glucose co-transporter 2 inhibitors

Table S2 Association of pre-stroke variables with renal dysfunction in male patients

Variables	Ccr <30 mL/min	Ccr ≥30 mL/min	P-value
	n=185	n=1720	
mRS before the acute stroke	1, 0–3	0, 0–1	<0.0001
Albumin, g/L	37, 33–40	41, 38–43	<0.0001
Hemoglobin, g/L	119, 107–128	142, 131–153	<0.0001
Glucose, mmol/L	7.05, 5.77–9.41	6.88, 5.83–8.62	0.2557
Glycated hemoglobin A1c, %	5.8, 5.4–6.45	5.9, 5.5–6.4	0.1243
LDL-C, mmol/L	2.42, 1.96–3.02	2.83, 2.27–3.43	<0.0001
HDL-C, mmol/L	1.28, 1.02–1.54	1.36, 1.12–1.66	0.0019
Triglyceride, mmol/L	1.00, 0.72–1.42	1.13, 0.79–1.80	0.0028
C-reactive protein, μg/L	3400, 1000–15500	1100, 500–3300	<0.0001
DPP4is, n (%)	34 (18.38%)	201 (11.69%)	0.0126
Biguanide, n (%)	19 (10.27%)	102 (5.93%)	0.0321
Alpha GIs, n (%)	13 (7.03%)	46 (2.67%)	0.0043
Insulin secretagogues, n (%)	9 (4.86%)	116 (6.74%)	0.3074
Long-acting insulin analog, n (%)	8 (4.32%)	43 (2.50%)	0.1748
SGLT2is, n (%)	1 (0.54%)	13 (0.76%)	1.0000
Loop diuretics, n (%)	55 (29.73%)	95 (5.52%)	<0.0001
Thiazide diuretics, n (%)	6 (3.24%)	34 (1.98%)	0.2856
MRAs, n (%)	12 (6.49%)	73 (4.24%)	0.1843
ARBs, n (%)	60 (32.43%)	387 (22.5%)	0.0034
L-type CCBs, n (%)	66 (35.68%)	366 (21.28%)	<0.0001
N-type or T-type CCBs, n (%)	10 (5.41%)	70 (4.07%)	0.4069
Verapamil, n (%)	7 (3.78%)	17 (0.99%)	0.0070
Diltiazem, n (%)	2 (1.08%)	17 (0.99%)	0.7064
ACEis, n (%)	9 (4.86%)	127 (7.36%)	0.1838
Alpha-blockers, n (%)	12 (6.49%)	23 (1.34%)	<0.0001
Alpha-beta blockers, n (%)	39 (21.08%)	126 (7.33%)	<0.0001
Beta-blockers, n (%)	21 (11.35%)	94 (5.47%)	0.0037
Statin, n (%)	44 (23.78%)	373 (21.69%)	0.5158
Warfarin, n (%)	22 (11.89%)	107 (6.22%)	0.0072
DOACs, n (%)	5 (2.7%)	99 (5.76%)	0.0576
Aspirin, n (%)	66(35.68%)	287 (16.69%)	<0.0001
Antiplatelets other than aspirin, n (%)	34 (18.38%)	210 (12.21%)	0.0229

All values except for categorical data are represented as median and interquartile ranges.

Alpha GIs, alpha glucosidase inhibitors; ACEis, angiotensin-converting enzyme inhibitors; ARBs, angiotensin receptor blockers; AS, acute stroke; CCBs, calcium channel blockers; Ccr, creatinine clearance; DOACs, direct-acting oral anticoagulants; DPP4is, dipeptidyl peptidase 4 inhibitors; HDL-C, high-density lipoprotein cholesterol; LDL-C, low-density lipoprotein cholesterol; MRAs, mineralocorticoid receptor antagonists; mRS, modified Rankin Scale; n, number; P, probability; SGLT2i, sodium-glucose co-transporter 2 inhibitors

Table S3 Association of pre-stroke variables with severe renal dysfunction in female patients

Variables	Ccr <30 mL/min	Ccr ≥30 mL/min	P-value
	n=293	n=1274	
mRS before the acute stroke	3, 0–4	0, 0–3	<0.0001
Albumin, g/L	37, 33–40	40, 37–43	<0.0001
Hemoglobin, g/L	117, 109–128	132, 121–140	<0.0001
Glucose, mmol/L	6.55, 5.61–8.05	6.66, 5.83–8.33	0.0607
Glycated hemoglobin A1c, %	5.7, 5.4–6.1	5.8, 5.5–6.2	0.0130
LDL-C, mmol/L	2.89, 2.22–3.64	3.24, 2.66–3.94	<0.0001
HDL-C, mmol/L	1.46, 1.16–1.83	1.58, 1.31–1.92	0.0001
Triglyceride, mmol/L	1.02, 0.77–1.39	1.05, 0.77–1.50	0.2262
C-reactive protein, μg/L	2200, 600–7850	1100, 400–4000	<0.0001
DPP4is, n (%)	22 (7.51%)	107 (8.40%)	0.6134
Biguanide, n (%)	9 (3.07%)	48 (3.77%)	0.5581
Alpha GIs, n (%)	4 (1.37%)	22 (1.73%)	0.6544
Insulin secretagogues, n (%)	14 (4.78%)	51 (4.00%)	0.5556
Long-acting insulin analog, n (%)	7 (2.39%)	18 (1.41%)	0.2539
SGLT2is, n (%)	0 (0%)	5 (0.39%)	0.5908
Loop diuretics, n (%)	83 (28.3%)	85 (6.67%)	<0.0001
Thiazide diuretics, n (%)	9 (3.07%)	25 (1.96%)	0.2611
MRAs, n (%)	49 (16.7%)	58 (4.6%)	<0.0001
ARBs, n (%)	88 (30.03%)	293 (23.0%)	0.0129
L-type CCBs, n (%)	76 (25.94%)	274 (21.51%)	0.1052
N-type or T-type CCBs, n (%)	14 (4.78%)	60 (4.71%)	0.9603
Verapamil, n (%)	8 (2.73%)	26 (2.04%)	0.4781
Diltiazem, n (%)	4 (1.37%)	12 (0.94%)	0.5188
ACEis, n (%)	21 (7.17%)	63 (4.95%)	0.1418
Alpha-blockers, n (%)	8 (2.73%)	17 (1.33%)	0.1089
Alpha-beta blockers, n (%)	39 (13.31%)	72 (5.65%)	<0.0001
Beta-blockers, n (%)	32 (10.92%)	79 (6.20%)	0.0071
Statin, n (%)	66 (22.53%)	266 (20.88%)	0.5364
Warfarin, n (%)	39 (13.31%)	69 (5.42%)	<0.0001
DOACs, n (%)	6 (2.05%)	67 (5.26%)	0.0100
Aspirin, n (%)	58 (19.8%)	154 (12.09%)	0.0009
Antiplatelets other than aspirin, n (%)	32 (10.92%)	99 (7.77%)	0.0887

All values except for categorical data are represented as median and interquartile ranges.

Alpha GIs, alpha glucosidase inhibitors; ACEis, angiotensin-converting enzyme inhibitors; ARBs, angiotensin receptor blockers; AS, acute stroke; CCBs, calcium channel blockers; Ccr, creatinine clearance; DOACs, direct-acting oral anticoagulants; DPP4is, dipeptidyl peptidase 4 inhibitors; HDL-C, high-density lipoprotein cholesterol; LDL-C, low-density lipoprotein cholesterol; MRAs, mineralocorticoid receptor antagonists; mRS, modified Rankin Scale; n, number; P, probability; SGLT2i, sodium-glucose co-transporter 2 inhibitors

Table S4 The cut-off value of hemoglobin for predicting Ccr <30 mL/min in male patients using receiver operating characteristics curve of logistic regression analysis

Variable	Sensitivity	Specificity	AUC
			0.853
Hemoglobin (≤130 vs >130) g/L	82.5%	76.7%	

AUC, area under the curve; Ccr, creatinine clearance

Figure S1 Receiver operating characteristics curve of hemoglobin for predicting Ccr <30 mL/min in male patients. Area under the curve is 0.853.

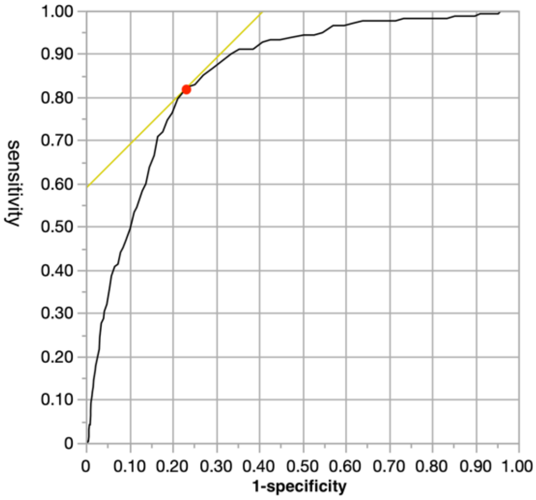

Table S5 The cut-off value of albumin for predicting Ccr <30 mL/min in male patients using receiver operating characteristics curve of logistic regression analysis

Variable	Sensitivity	Specificity	AUC
			0.735
Albumin (≤38 vs >38) g/L	67.6%	69.2%	

AUC, area under the curve; Ccr, creatinine clearance

Figure S2 Receiver operating characteristics curve of albumin for predicting Ccr <30 mL/min in male patients. Area under the curve is 0.735.

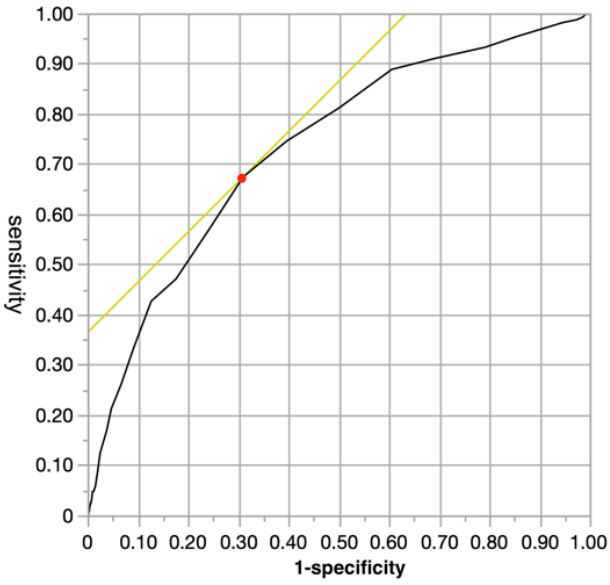

Table S6 The cut-off value of hemoglobin for predicting Ccr <30 mL/min in female patients using receiver operating characteristics curve of logistic regression analysis

Variable	Sensitivity	Specificity	AUC
			0.727
Hemoglobin (≤124 vs >124) g/L	66.7%	67.5%	

AUC, area under the curve; Ccr, creatinine clearance

Figure S3 Receiver operating characteristics curve of hemoglobin for predicting Ccr <30 mL/min in female patients. Area under the curve is 0.7

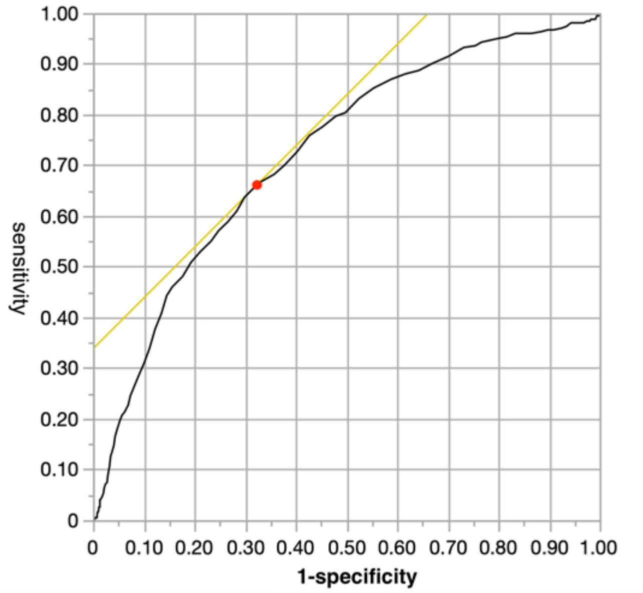

Table S7 Association of loop diuretic use with other heart-disease relevant medications in male patients: the contingency table analysis

Variables			P-value
	Aspirin		<0.0001
Loop diuretics	Use	No use	
Use, n (%)	55 (36.7%)	95 (63.3%)	
No use, n (%)	298 (17.0%)	1457 (83.0%)	
	MRAs		<0.0001
loop diuretics	Use	No use	
Use, n (%)	102 (68.0%)	48 (32.0%)	
No use, n (%)	37 (2.11%)	1718 (97.9%)	
	L-type CCB		0.0752
Loop diuretics	Use	No use	
Use, n (%)	43 (28.7%)	107 (71.3%)	
No use, n (%)	389 (22.2%)	1366 (77.8%)	
	Alpha blockers		0.4574
loop diuretics	Use	No use	
Use, n (%)	4 (2.67%)	146 (97.3%)	
No use, n (%)	31 (1.77%)	1724 (98.2%)	
	Alpha-beta blockers		<0.0001
Loop diuretics	Use	No use	
Use, n (%)	45 (30.0%)	105 (70.0%)	
No use, n (%)	120 (6.8%)	1635 (93.2%)	
	Beta blockers		<0.0001
Loop diuretics	Use	No use	
Use, n (%)	25 (16.7%)	125 (83.3%)	
No use, n (%)	90 (5.1%)	1665 (94.9%)	
	ARBs		0.0001
Loop diuretics	Use	No use	
Use, n (%)	55 (36.7%)	95 (63.3%)	
No use, n (%)	392 (22.3%)	1363 (77.7%)	
	ACEi		0.0052
Loop diuretics	Use	No use	
Use, n (%)	20 (13.3%)	130 (86.7%)	
No use, n (%)	116 (6.6%)	1639 (93.4%)	
	DOACs		0.0084
Loop diuretics	Use	No use	
Use, n (%)	16 (10.7%)	134 (89.3%)	
No use, n (%)	88 (5.0%)	1667 (95.0%)	
	Warfarin		<0.0001
Loop diuretics	Use	No use	
Use, n (%)	32 (21.3%)	118 (78.7%)	
No use, n (%)	97 (5.5%	1658 (94.5%)	

ACEis, angiotensin-converting enzyme inhibitors; ARBs, angiotensin receptor blockers; CCBs, calcium channel blockers; DOACs, direct-acting oral anticoagulants; MRAs, mineralocorticoid receptor antagonists; P, probability

Table S8 Association of triple-whammy with renal function in male patients

Variables		Ccr <30 mL/min	Ccr ≥30 mL/min	P-value
Triple whammy_ARB				<0.0001
	Yes, n (%)	13 (52%)	12 (48%)	
	No, n (%)	172 (9.1%)	1708 (90.9%)	
Triple whammy_ACE				0.3278
	Yes, n (%)	2 (16.7%)	10 (83.3%)	
	No, n (%)	183 (9.7%)	1710 (90.3%)	
Triple whammy_thiazide				1.0000
	Yes, n (%)	0 (0%)	4 (100%)	
	No, n (%)	185 (9.7%)	1716 (90.3%)	

ACEis, angiotensin-converting enzyme inhibitors; ARBs, angiotensin receptor blockers; Ccr, creatinine clearance; P, probability; Triple whammy_ARB, ARBs + aspirin + loop diuretics; Triple whammy_ACE, ACEi + aspirin + loop diuretics; triple whammy_thiazide, (ACEi or ARB) + asoirin + thiazide diuretics

Table S9 Association of loop diuretic use with other heart-disease relevant medications in female patients: the contingency table analysis

variables			P-value
	Aspirin		<0.0001
Loop diuretics	Use	No use	
Use, n (%)	47 (28.0%)	121 (72.0%)	
No use, n (%)	165 (11.8%)	1235 (88.2%)	
	MRAs		<0.0001
Loop diuretics	Use	No use	
Use, n (%)	60 (36.7%)	108 (64.3%)	
No use, n (%)	46 (3.3%)	1354 (96.7%)	
	L-type CCB		0.7641
Loop diuretics	Use	No use	
Use, n (%)	36 (21.4%)	132 (78.6%)	
No use, n (%)	314 (22.4%)	1086 (77.6%)	
	Alpha-beta blockers		<0.0001
Loop diuretics	Use	No use	
Use, n (%)	39 (23.2%)	129 (76.8%)	
No use, n (%)	73 (5.2%)	1327 (94.8%)	
	Beta blockers		<0.0001
Loop diuretics	Use	No use	
Use, n (%)	28 (16.7%)	140 (83.3%)	
No use, n (%)	83 (5.9%)	1317 (94.1%)	
	ARBs		0.0382
Loop diuretics	Use	No use	
Use, n (%)	52 (31.0%)	116 (69.0%)	
No use, n (%)	329 (23.5%)	1071 (76.5%)	
	ACEi		0.4822
Loop diuretics	Use	No use	
Use, n (%)	11 (6.6%)	157 (93.4%)	
No use, n (%)	73 (5.2%)	1327 (94.8%)	
	DOACs		0.0045
Loop diuretics	Use	No use	
Use, n (%)	16 (9.5%)	152 (90.5%)	
No use, n (%)	57 (4.1%)	1343 (95.9%)	
	Warfarin		<0.0001
Loop diuretics	Use	No use	
Use, n (%)	37 (22.0%)	131 (78.0%)	
No use, n (%)	72 (5.1%)	1328 (94.9%)	

ACEis, angiotensin-converting enzyme inhibitors; ARBs, angiotensin receptor blockers; CCBs, calcium channel blockers; DOACs, direct-acting oral anticoagulants; MRAs, mineralocorticoid receptor antagonists; P, probability

Table S10 Association of triple-whammy with renal function in female patients

variables	Ccr <30 mL/min	Ccr ≥30 mL/min	P-value
Triple whammy_ARB			0.0025
Yes, n (%)	8 (53.3%)	7 (46.7%)	
No, n (%)	285 (18.4%)	1267 (81.6%)	
Triple whammy_ACE			0.4628
Yes, n (%)	1 (33.3%)	2 (66.7%)	
No, n (%)	292 (18.7%)	1272 (81.3%)	
Triple whammy_thiazide			1.0000
Yes, n (%)	0 (0%)	3 (100%)	
No, n (%)	293 (18.7%)	1271 (81.3%)	

ACEis, angiotensin-converting enzyme inhibitors; ARBs, angiotensin receptor blockers; Ccr, creatinine clearance; P, probability; Triple whammy_ARB, ARBs + aspirin + loop diuretics; Triple whammy_ACE, ACEi + aspirin + loop diuretics; triple whammy_thiazide, (ACEi or ARB) + asoirin + thiazide diuretics
